# Supplementary material for: Asynchronous magnetic resonance elastography: Shear wave speed reconstruction using noise correlation of incoherent waves
Source: Magn Reson Med. 2022 Oct 27;89(3):990–1001. doi: 10.1002/mrm.29502 (PMC9792433; doi:10.1002/mrm.29502)
Supplement: Supplementary file 1 — DATA S1 MATLAB code used to generate the numerical simulations of Figure 1 [file MRM-89-990-s002.zip › k-Wave/helpfiles/example_ivp_saving_movie_files.html]

Saving Movie Files Example (k-Wave)


# Saving Movie Files Example

This example demonstrates how to save the simulation animations as a movie. It builds on the Heterogeneous Propagation Medium Example.

- Open the file in the MATLAB Editor
- Run the file in MATLAB

## Contents

- Setting the 'RecordMovie' flag
- Controlling the movie settings
- Changing additional display settings

## Setting the 'RecordMovie' flag

In the preceding example, the optional inputs `'PlotLayout'` and `'PlotPML'` were used to change the default behaviour of `kspaceFirstOrder2D`. Here several other optional inputs are used to save the simulation animation to a movie file. By setting `'RecordMovie'` to `true`, the displayed image frames are saved and exported as a movie with a date and time stamped filename. A user defined filename can also be given by setting the `'MovieName'` input to a string. The movie frames are captured using `getframe` and converted to a video file using `VideoWriter`. Note, when using `getframe`, if other windows are moved in front of the simulation window, these may also be captured.

```
% set the input arguments
input_args = {'RecordMovie', true, 'MovieName', 'example_movie'};

% run the simulation
sensor_data = kspaceFirstOrder2D(kgrid, medium, source, sensor, input_args{:});
```

## Controlling the movie settings

As the movie frames mimic the frames used for the simulation animation, the properties of the movie can be controlled by changing the animation settings. For example, the image scaling can be set using `'PlotScale'`, and the number of iterations which must pass before the simulation plot is updated can be controlled via `'PlotFreq'`. Note, the default color map used for the animation has zero set to white (see `getColorMap`), thus using a plot scaling parameter set to `[-a, a]` will give the best visual results.

```
% set the input arguments
input_args = {..., 'PlotScale', [-2, 2], 'PlotFreq', 5, ...};
```

The profile and properties of `VideoWriter` can also be modified via the optional inputs `'MovieProfile'` and `'MovieArgs'`. An example of changing the default frame rate, and saving the movie as an MP4 is given below.

```
% set the input arguments
input_args = {..., 'MovieProfile', 'MPEG-4', 'MovieArgs', {'FrameRate', 10}, ...};
```

The movies are saved in the same directory as the example m-file.

Note, by default, k-Wave sets `'MovieProfile'` to `'Uncompressed AVI'`, which means the generated movie files can become very large. If using a recent version of MATLAB, it's recommended to use the optional input `'MovieProfile', 'MPEG-4'`. Alternatively, to reduce the size after the movie is created, the open source package Hand Brake can be used to compress and covert the files.

## Changing additional display settings

A wide range of different visualisations can be produced by modifying additional input parameters.
For example, a mesh plot can be produced instead of an image plot by setting `'MeshPlot'` to `true` (supported in 2D only), the display mask can be customised or switched off using `'DisplayMask'`, and the visibility of the PML can be controlled using `'PlotPML'`.
If no sensor output is required, the sensor input can also be left blank using an empty array.

```
% set the input arguments
input_args = {..., 'MeshPlot', true, 'DisplayMask', 'off', 'PlotPML', false, ...};

% run the simulation without a sensor input
kspaceFirstOrder2D(kgrid, medium, source, [], input_args{:});
```

Note, if you see a blank movie when using the option `'MeshPlot'` set to `true`, this can sometimes be solved by running the command `opengl('software')`. This forces MATLAB to use software OpenGL rendering instead of hardware OpenGL.
